# Lyme Disease Patient Trajectories Learned from Electronic Medical Data for Stratification of Disease Risk and Therapeutic Response

**DOI:** 10.1038/s41598-019-41128-x

**Published:** 2019-03-14

**Authors:** Osamu Ichikawa, Benjamin S. Glicksberg, Nicholas Genes, Brian A. Kidd, Li Li, Joel T. Dudley

**Affiliations:** 1 Department of Genetics and Genomic Sciences, Institute for Next Generation Healthcare, Icahn School of Medicine at Mount Sinai, One Gustave L. Levy Place Box 1498, New York, NY 10029 USA; 2 Drug Research Division, Sumitomo Dainippon Pharma. Co. Ltd., 3-1-98 Kasugade-naka, Konohana-ku, Osaka, 554-0022 Japan; 3 Bakar Computational Health Science Institute, University of California, 550 16th St, San Francisco, California 94158 USA; 4 Department of Emergency Medicine, Icahn School of Medicine at Mount Sinai, One Gustave L. Levy Place, 1190 Fifth Avenue Box 1620, New York, NY 10029 USA; 5 Sema4, a Mount Sinai Venture, Stamford, Connecticut 06902 USA

## Abstract

Lyme disease (LD) is the most common tick-borne illness in the United States. Although appropriate antibiotic treatment is effective for most cases, up to 20% of patients develop post-treatment Lyme disease syndrome (PTLDS). There is an urgent need to improve clinical management of LD using precise understanding of disease and patient stratification. We applied machine-learning to electronic medical records to better characterize the heterogeneity of LD and developed predictive models for identifying medications that are associated with risks of subsequent comorbidities. For broad disease categories, we identified 3, 16, and 17 comorbidities within 2, 5, and 10 years of diagnosis, respectively. At a higher resolution of ICD-9 codes, we identified known associations with LD including chronic pain and cognitive disorders, as well as particular comorbidities on a timescale that matched PTLDS symptomology. We identified 7, 30, and 35 medications associated with risks of these comorbidities within 2, 5, and 10 years, respectively. For instance, the first-line antibiotic doxycycline exhibited a consistently protective association for typical symptoms of LD, including backache. Our approach and findings may suggest new hypotheses for more personalized treatments regimens for LD patients.

## Introduction

Lyme disease (LD) is a vector-borne, infectious disease caused by the bacterium *Borrelia burgdorferi* that is transmitted to humans through tick bites. According to the US Centers for Disease Control and Prevention (CDC), around 329,000 LD cases occur annually^1^ and it becomes a major US public health problem that causes substantial use of health care resources. LD is most prevalent in the Northeast and upper Midwest, and 95% of all confirmed cases in 2015 were reported in 14 states^2^. The symptomology of LD is heterogeneous, although some general patterns have emerged. The first manifestation of LD is often an expanding annular lesion, called erythema migrans, near the bite location, but this sign is present in only 70–80% of patients^3^. The length of time for the rash to occur, along with the characteristics of the rash (e.g., composition and size) can also vary^4^. Other clinical features that often arise, singly or in combination, include fever, pain, arthritis, neurological symptoms (e.g., facial nerve palsy), and satellite rashes. One explanation for the variability in Lyme Disease (LD) symptoms is that the genotype of the *Borrelia burgdorferi* itself might affect aspects of pathogenesis, such as the probability of hematogenous dissemination^5,6^. The neurological manifestations in LD, reported in 3–12% of patients, are of great concern^7^. These phenomena, collectively called neuroborreliosis, are often associated with intense pain that can manifest either soon after infection or much later, from months to years afterward.

Accurate and precise diagnoses of LD present several challenges. Typically, laboratory testing of LD follows identification of cutaneous manifestations from visual inspection but these manifestations are not always present (see Supplementary Background for further exploration of issues regarding laboratory testing for LD). Many studies have attempted to develop methods for differentiating LD from other similar syndromes, e.g., septic arthritis vs. LD of the knee in children^8^.

Following successful diagnosis, LD is most commonly treated with antibiotics such as doxycycline, amoxicillin, cefuroxime axetil, and ceftriaxone. Although these medications have high cure rates (~90%)^9^, they are associated with serious complications and adverse events, especially under prolonged use^4,10–14^. One study even showed that certain first-line treatments, specifically intravenous ceftriaxone followed by doxycycline for chronic symptoms in LD, were not effective compared to placebo forcing discontinuation of the trial^13^. Another study reported that repeated IV ceftriaxone treatment for Lyme encephalopathy resulted in only minor cognitive improvements, with high rates of relapse of cognitive symptoms^14^. These findings suggest that unknown factors are responsible for the high variability of treatment outcomes for patients with disseminated LD. Additionally, up to 20% of treated patients develop post-treatment Lyme disease syndrome (PTLDS), in which lingering symptoms such as fatigue, pain, or joint and muscle aches last for months or even years. The causes and frequencies of these symptoms remain unclear, and the issue is further confounded by the presence of concurrent diseases.

It is difficult to disentangle to what extent given treatment responses and disease sequelae are due to differences in individual immune responses, patient characteristics, disease burden, and treatment timing, or to the medications themselves. Indeed, it is very likely that response and outcome depend on a complex interplay between these factors, making clinicians’ jobs extremely difficult^15,16^. To address the diverse symptomology, imperfect diagnostic strategies (see Supplementary Background), and variable treatment outcomes of LD, comprehensive study designs are required.

Although the aforementioned studies have provided a great deal of useful information, the variability in global risk profiles for LD pathogenesis remains incompletely understood, and there is an unmet need for personalized treatment recommendations that take into account individual characteristics such as demographics and disease burden. Electronic Medical Records (EMRs) from hospitals contain a wealth of longitudinal, patient-level data encompassing prior history of prescriptions and disease diagnoses, along with clinical outcomes, that can be exploited to investigate these issues in a data-driven fashion PMID: 29659828. To date there has been no systematic analysis of LD using EMR data, particularly from a hospital within a high-risk state (see Supplementary Background).

**Figure 1:**
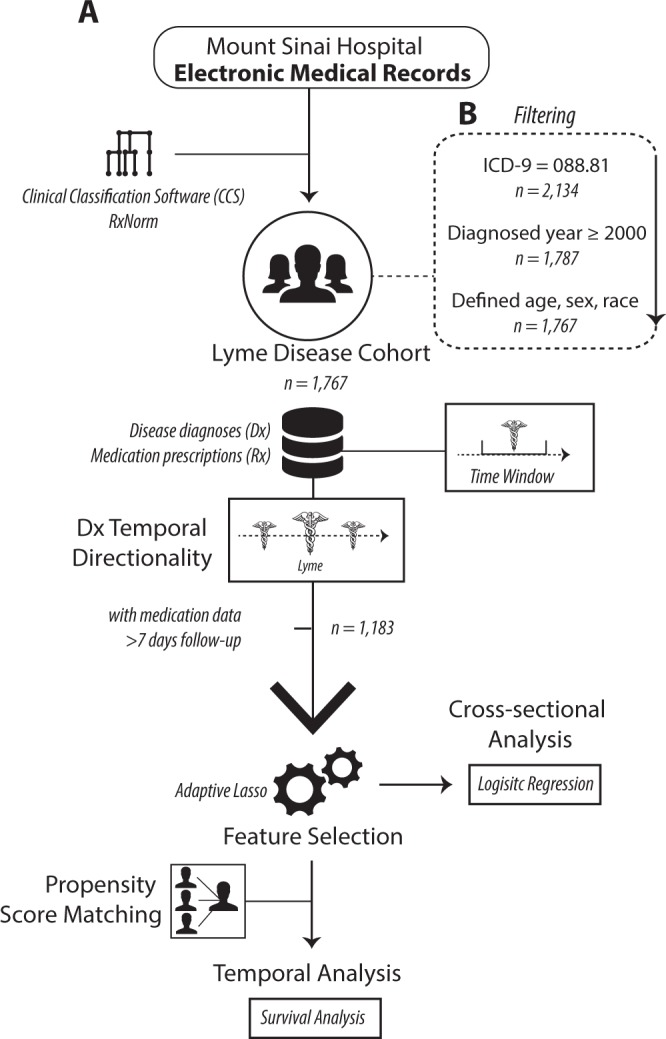
Workflow of the study, outlining steps from data organization to statistical methodologies.

We hypothesized that EMR data from Mount Sinai Hospital in New York City could provide a rich framework for studying the heterogeneity of Lyme manifestation, as well as the quality and efficacy of treatment. Using various state-of-the-art statistical and machine learning methods, our study is the first data-driven effort to prioritize medications for LD based on an individual’s phenotype profile. We identified Lyme-associated comorbidities at the level of broad disease categories, pinpointed specific co-morbid diseases associated with LD over time, including the diseases known to follow LD, and we predicted medications that associate with risk of developing these comorbidities (Fig. 1). We expect that the novel framework and findings from this study can be used to support future efforts to develop personalized treatment strategies for patients with LD, including providing physicians with a broader evidentiary foundation on which to base their treatment recommendations (e.g., selection of antibiotics) based on individual patients’ disease background.

## Results

### Identified comorbidities associated with Lyme disease, grouped as broader disease categories

We assessed the temporal ordering of the associations between Lyme disease (LD) and other diseases (grouped by CCS category) to determine whether a given comorbidity tended to occur before or after diagnosis of LD. We categorized diseases using the Clinical Classifications Software (CCS) for ICD-9 diagnosis codes, developed by AHRQ 14, which aggregates and characterizes more than 14,000 ICD-9 codes into broader coherent 283 disease categories (Supplemental Methods). This strategy helps to avoid sample size limitation as a result of using ICD-9 codes alone. We restricted our analysis to diseases with reported dates and only included the first reported encounter of a diagnosis and analyzed their temporal ordering at the patient level. For each comorbid disease pair (i.e., LD and another disease category), we tabulated the number of patients with both diseases and assessed which disease in the pair occurred first, or if they occurred at the same time, based on the visit dates. Out of the 275 Lyme-comorbidity combinations (restricting to those with at least 20 affected patients of both conditions) for all time windows, 21 were nominally significant, with 5 diseases occurring prior to LD and 16 occurring after (*p* < 0.1 due to the relatively small sample size; Table 1). For the 2-year window, we identified three disease categories significantly associated with LD, with two prior and one after; for the 5-year window, 16 categories, with four prior and 12 after; and for the 10-year window, 17 categories with five prior and 12 after (Table 1). We confirmed some reported Lyme comorbidities, including ‘nutritional deficiencies’^17,18^, ‘vision defect’^19,20^, and ‘disorder of lipid metabolism’^21^. Additionally, we identified several disease comorbidities not previously reported, including ‘cataract’, ‘acute bronchitis’ and ‘nonmalignant breast conditions’. A complete list of disease categories is shown in Table 1.

**Table 1:** Diseases associated with Lyme, analyzed as CCS-single-level categories (p value < 0.1).

Time (year)	Direction	Disease (CCS)	Lyme First	Disease First	Same Time	P value	Prob.
2	—	Coronary atherosclerosis and other heart disease	18	58	17	1.10E-02	0.62
2	—	Administrative/social admission	41	66	9	8.17E-02	0.57
2	+	Acute bronchitis	23	11	3	9.39E-02	0.62
5	—	HIV infection	2	21	9	5.51E-02	0.66
5	—	Coronary atherosclerosis and other heart disease	38	80	17	1.92E-02	0.59
5	—	Administrative/social admission	55	88	9	3.09E-02	0.58
5	—	Disorders of lipid metabolism	117	269	111	3.63E-02	0.54
5	+	Open wounds of extremities	24	6	0	7.15E-04	0.8
5	+	Open wounds of head; neck; and trunk	19	9	0	4.36E-02	0.68
5	+	Fracture of lower limb	16	8	0	7.58E-02	0.67
5	+	Cataract	57	21	10	3.67E-03	0.65
5	+	Fracture of upper limb	18	9	1	9.25E-02	0.64
5	+	Acute bronchitis	36	17	3	2.20E-02	0.64
5	+	Anal and rectal conditions	26	11	5	8.21E-02	0.62
5	+	Nonmalignant breast conditions	60	36	7	5.72E-02	0.58
5	+	Other eye disorders	75	41	14	4.76E-02	0.58
5	+	Neoplasms of unspecified nature or uncertain behavior	60	39	6	8.58E-02	0.57
5	+	Blindness and vision defects	58	33	11	9.89E-02	0.57
5	+	Nutritional deficiencies	219	141	47	6.85E-02	0.54
10	—	HIV infection	2	25	9	1.44E-02	0.69
10	—	Coronary atherosclerosis and other heart disease	44	91	17	9.18E-03	0.6
10	—	Administrative/social admission	61	97	9	2.20E-02	0.58
10	—	Disorders of lipid metabolism	121	301	111	1.59E-03	0.56
10	—	Essential hypertension	96	271	124	1.20E-02	0.55
10	+	Open wounds of extremities	28	7	0	2.54E-04	0.8
10	+	Maintenance chemotherapy; radiotherapy	14	5	1	5.77E-02	0.7
10	+	Cataract	64	26	10	3.32E-03	0.64
10	+	Occlusion or stenosis of precerebral arteries	22	10	3	8.77E-02	0.63
10	+	Acute bronchitis	37	19	3	3.37E-02	0.63
10	+	Poisoning by other medications and drugs	24	7	8	9.98E-02	0.62
10	+	Anal and rectal conditions	29	14	5	9.67E-02	0.6
10	+	Other eye disorders	87	43	14	7.69E-03	0.6
10	+	Nonmalignant breast conditions	65	41	7	6.60E-02	0.58
10	+	Blindness and vision defects	63	36	11	7.62E-02	0.57
10	+	Inflammation; infection of eye (except that caused	78	39	20	6.19E-02	0.57
		by tuberculosis or sexually transmitted disease)					
10	+	Nutritional deficiencies	228	143	47	3.51E-02	0.55

A total of 275 diseases were tested.

### Highlighted specific known or novel diseases associated with LD, analyzed at higher resolution

Although the CCS broad categories were helpful in identifying disease groups of relevance from a broader perspective, we also performed the same analysis at a higher granularity (Table 2 and Supplementary Table 1). To this end, using the ICD-9 codes, we sought to determine which specific diseases drove the signal and whether the signal still persisted. A total of 3,639 Lyme–comorbidity combinations were analyzed using the ICD-9 codes. We identified known associations that occur within a 10-year window after LD (p < 0.1 due to the relatively small sample size). We found associations relating to pain, including ‘chronic pain Not Elsewhere Classifiable (NEC)’ (p = 0.015), ‘joint pain-shoulder (shoulder)’ (p = 0.047), ‘pain in limb’ (p = 0.074), ‘throat pain’ (p = 0.001), and ‘tension headache Not Otherwise Specified (NOS)’ (p = 0.063) (Supplementary Table 1). We also identified associations related to cognitive issues, specifically ‘dementia NOS w behavioral (behav) disturbance (distrb)’ (p = 0.016), ‘dementia NOS w/o behavioral (behv) disturbance (dstrb)’ (p = 0.006), and ‘Alzheimer’s disease’ (p = 0.073).

**Table 2:** Diseases associated with Lyme, by ICD-9 code. A total of 3,639 diseases were tested.

Time (year)	Direction	Disease (CCS)	ICD9	Disease (ICD9)	Lyme First	Disease First	Same Time	p value (binomial)	Prob. (binomial)	p value (logistic regression)	OR (logistic regression)
5	—	HIV infection	V08	HIV positive NOS	0	21	8	1.21E-02	0.72	1.80E-05	2.17
5	—	Administrative/social admission	V20.2	Routin child health exam	16	49	4	3.18E-04	0.71	1.31E-02	2.08
5	+	Nutritional deficiencies	268.9	Vitamin D deficiency NOS	203	124	36	1.37E-02	0.56	1.20E-177	5.64
5	+	Cataract	366.9	Cataract NOS	40	18	7	4.08E-02	0.62	2.90E-30	4.27
5	+	Acute bronchitis	466.0	Acute bronchitis	36	16	3	1.50E-02	0.65	4.02E-21	3.56
5	+	Nonmalignant breast conditions	793.80	Unspecified abnormal mammogram	28	12	0	8.29E-03	0.70	8.92E-10	2.61
10	—	HIV infection	V08	HIV positive NOS	0	24	8	3.50E-03	0.75	1.80E-05	2.17
10	—	Disorders of lipid metabolism	272.0	Pure hypercholesterolem	84	159	50	8.04E-02	0.54	1.60E-30	2.22
10	—	Disorders of lipid metabolism	272.4	Hyperlipidemia NEC/NOS	97	187	64	9.01E-02	0.54	1.06E-18	1.78
10	—	Essential hypertension	401.9	Hypertension NOS	96	247	120	8.16E-02	0.53	9.83E-08	1.39
10	—	Administrative/social admission	V20.2	Routin child health exam	17	54	4	8.82E-05	0.72	1.31E-02	2.08
10	+	Nutritional deficiencies	268.9	Vitamin D deficiency NOS	211	124	36	4.67E-03	0.57	1.20E-177	5.64
10	+	Cataract	366.16	Senile nuclear cataract	17	2	1	1.29E-03	0.85	2.94E-03	1.97
10	+	Cataract	366.9	Cataract NOS	45	21	7	3.02E-02	0.62	2.90E-30	4.27
10	+	Inflammation; infection of eye (except that caused by tuberculosis or sexually transmitted disease)	373.00	Blepharitis NOS	33	2	1	1.14E-07	0.92	2.27E-14	3.79
10	+	Other eye disorders	375.15	Tear film insuffic NOS	24	11	1	3.26E-02	0.67	4.44E-12	3.36
10	+	Acute bronchitis	466.0	Acute bronchitis	37	17	3	1.66E-02	0.65	4.02E-21	3.56
10	+	Nonmalignant breast conditions	793.80	Unspecified abnormal mammogram	31	12	0	2.70E-03	0.72	8.92E-10	2.61

This table includes only the ICD-9 diseases that are classified into the CCS-single-level categories shown in (a), and a full list of associations is provided in Supplementary Table 1.

At the 2-year window, five pairs were nominally significant, with four prior to LD and one after (Supplementary Table 1). For the 5-year window, we found 53 significant associations, with 49 prior to LD and four afterwards. For the 10-year window, we found 75 significant associations, with 67 prior to LD diagnosis and eight after. The significance of all disease categories significantly associated with LD that we identified in the previous ICD-level analysis persisted, including the four diseases that significantly occurred prior to LD: ‘pure hypercholesterolemia’ (p = 0.080 at 10 years), ‘hyperlipidemia NEC/NOS’ (p = 0.090 at 10 years), ‘hypertension NOS’ (p = 0.082 at 10 years), and ‘coronary atherosclerosis (athero) NOS’ (p = 0.022 at 5 years; p = 0.075 at 10 years). Nine sequelae diseases of are particular interest, specifically: ‘vitamin D deficiency NOS’ (p = 0.014 at 5 years; p = 0.0047 at 10 years), ‘cataract NOS’ (p = 0.041 at 5 years; p = 0.030, prob = 0.62 at 10 years), ‘senile nuclear cataract’ (p = 0.0013 at 10 years), ‘tear film insufficiency (insuffic) NOS’ (p = 0.033 at 10 years), ‘acute bronchitis’ (p = 0.015 at 5 years; p = 0.017 at 10 years) and ‘HIV positive NOS’ (p = 0.012 at 5 years; p = 0.0035 at 10 years) drove the signal from the broad disease categories (Table 2).

We confirmed the large majority of the comorbidity pairs were significantly associated with LD with concordant directionality by adjusting age, gender, and race by logistic regression (p < 0.1). Many of these include known associations mentioned above. We provide a complete list of ICD-9 level disease associations that passed our significance threshold in both analyses (Supplementary Table 1).

### Medications predicted to modulate risk of subsequent comorbidities in LD patients, analyzed as broader disease categories

**Figure 2:**
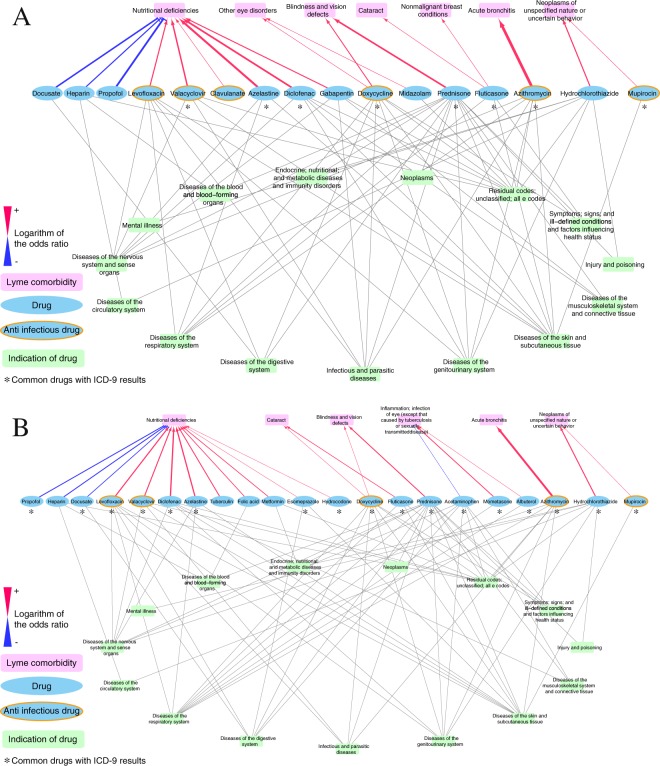
Medication–Lyme disease comorbidity network, analyzed by CCS-single-level categories, in time windows of 5 years (**A**) and 10 years (**B**). Significant associations between medications (cyan) and comorbidities (magenta) are connected by red or blue lines (p < 0.1). Red lines indicate risk associations (OR > 1), and blue lines indicate protective associations (OR < 1). Medications and indications (green) were connected based on information in the public knowledgebase MEDI^44^.

To investigate associations between prescribed medications and development of subsequent disease pathogenesis, we focused on comorbidities with onset after the first diagnosis of LD. Using the adaptive LASSO methodology and a logistic regression model, we investigated all medications prescribed to LD patients prior to the comorbidities. We found 3, 12, and 18 medications associated with disease comorbidities, classified by CCS-single-level categories, within 2, 5, and 10 years after Lyme diagnosis, respectively (Supplementary Table 2, Fig. 2A,B). Four medication–LD comorbidity associations were supported by published studies^22–26^,and we confirmed that these medications modulated the risks of Lyme comorbidities, including fluticasone–‘cataract’ (adjusted OR = 1.94, p = 0.072 at 5 years; adjusted OR = 2.01, p = 0.033 at 10 years) hydrochlorothiazide–‘neoplasms of unspecified nature or uncertain behavior’ (adjusted OR = 2.23, p = 0.031 at 5 years; adjusted OR = 2.48, p = 0.0092 at 10 years), metformin–‘nutritional deficiencies’ (adjusted OR = 2.05, p = 0.097 at 10 years), and esomeprazole–‘nutritional deficiencies’ (adjusted OR = 1.75, p = 0.093 at 10 years).

Five antibiotics, doxycycline, azithromycin, levofloxacin, clavulanate, and mupirocin, and one antiviral drug, valacyclovir, were predicted to modulate the risk of subsequent comorbidities. Doxycycline, a first-line antibiotic that was the most prescribed antibiotic in our EMR for patients with LD (39%, N = 553), was associated with an elevated risk of eye disorders, including ‘cataract’ (adjusted OR = 2.05, p = 0.092 at 2 years; adjusted OR = 1.70, p = 0.067 at 10 years), ‘blindness and vision disorders’ (adjusted OR = 2.05, p = 0.016 at the 5 years; adjusted OR = 1.95, p = 0.019 at 10 years), and ‘other eye disorders’ (adjusted OR = 1.81, p = 0.024) (Fig. 2A).

In regard to ‘nutritional deficiencies’, 11 medications were predicted to be risk factors and three to be protective. Among the 11 risk factor medications were two antibiotics, levofloxacin (adjusted OR = 2.26, p = 0.0093 at 5 years; adjust OR = 2.77, p = 7.0E-4 at 10 years) and clavulanate (adjusted OR = 1.64, p = 0.094 at 10 years), and one antiviral prophylactic, valacyclovir (adjusted OR = 2.56, p = 0.014 at 5 years; adjusted OR = 2.58, p = 0.011 at 10 years). Interestingly, we could identify potential new therapeutic options for the LD adjunctive treatment that warrant further study and replication analyses. Three medications, propofol, docusate, and heparin, consistently were associated with decreased risk of ‘nutritional deficiencies’ at 5 and 10 years after LD (Fig. 2). In addition, acetaminophen exhibited a potential protective effect at the early stage (2 years post-Lyme) (Supplementary Table 2).

### Medications predicted to modulate risk of subsequent comorbidities in LD patients, analyzed at the ICD-9 level

**Figure 3:**
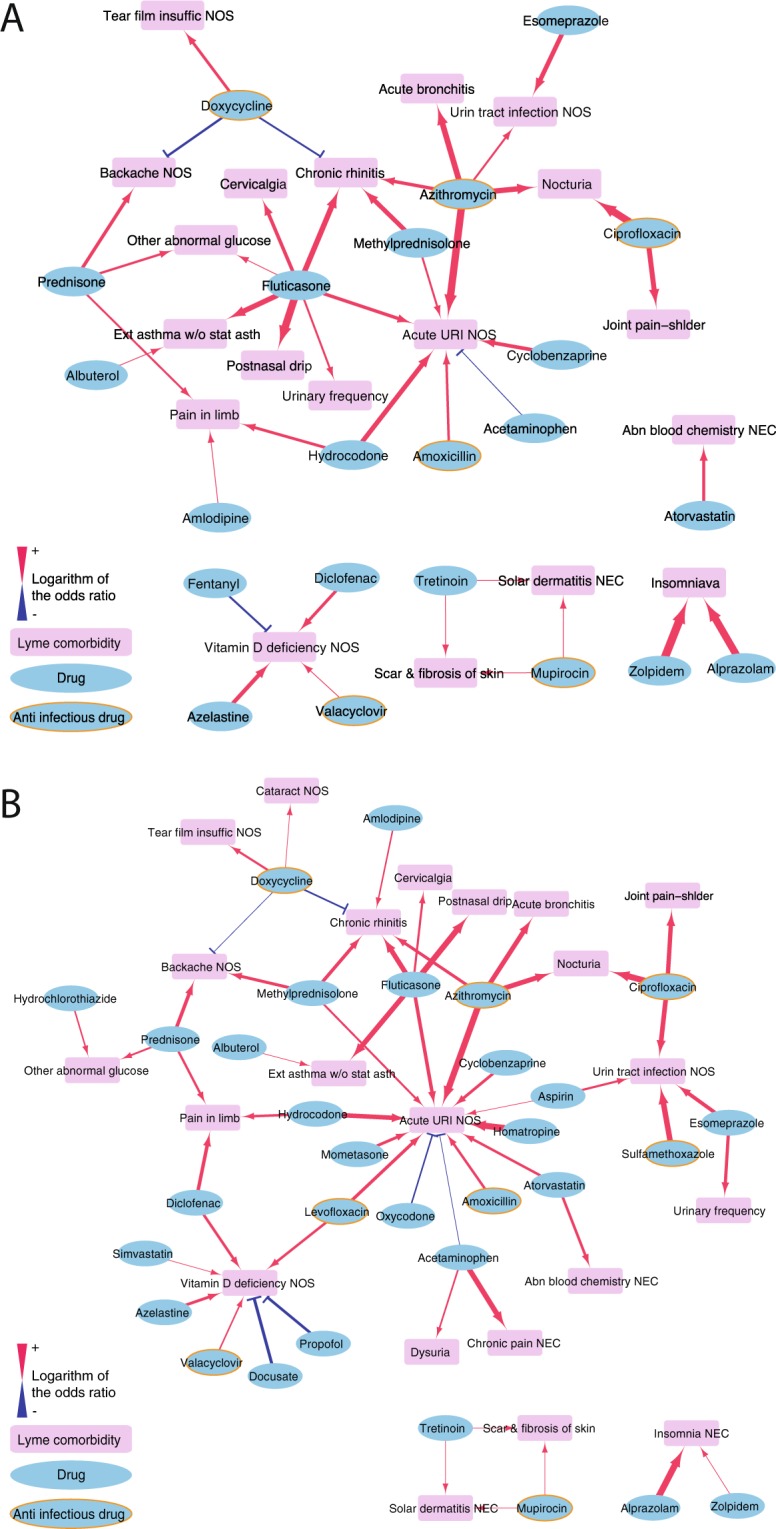
Medication–Lyme disease comorbidity network at the ICD-9 levels in time windows of 5 years (**A**) and 10 years (**B**). Significant associations between medications (cyan) and comorbidities (magenta) are connected by red or blue lines (p < 0.1). Red lines indicate risk associations (OR > 1), and blue lines indicate protective associations (OR < 1).

In the higher-resolution analysis using ICD-9 codes, we identified 7, 22, and 31 medications that were significantly associated with the disease comorbidities at 2, 5, and 10 years post-LD (Supplementary Table 3, Fig. 3A,B). Seven were previously reported risk associations including steroid prednisone was a risk for ‘pain in limb’, ciprofloxacin was a risk for ‘joint pain-shoulder (shlder)’^27–30^, and five of the side effects for four medications were reported in the SIDER database^31,32^ (Supplementary Table 3).

Two steroids, fluticasone and mometasone, and one pain reliever, hydrocodone, were associated with increased risk for ‘acute upper respiratory infection (URI) NOS’ in comparison with the placebo group (Supplementary Table 3) and were rediscovered in our study (respectively: adjusted OR = 2.92, p = 5.2E-5 at 5 years/adjusted OR = 3.44, p = 4.7E-7 at 10 years; adjusted OR = 2.86, p = 0.0028 at 10 years; adjusted OR = 4.01, p = 4.1E-5 at 5 years/adjusted OR = 4.45, p = 1.8E-6 at 10 years). We also reconfirmed the risk associations between fluticasone and ‘chronic rhinitis’ (adjusted OR = 4.70, p = 1.4E-4 at 2 years/adjusted OR = 4.78, p = 6.5E-7 at 5 years/adjusted OR = 4.86, p = 4.4E-8 at 10 years) and diclofenac and ‘pain in limb’ (adjusted OR = 3.43, p = 0.0011 at 10 years).

Doxycycline exhibited a consistently protective effect against ‘backache NOS’ (adjusted OR = 0.44, p = 0.018 at 5 years/adjusted OR = 0.50, p = 0.035 at 10 years), which is a typical symptom of LD, and ‘chronic rhinitis’ (adjusted OR = 0.48, p = 0.036 at 5 years/adjusted OR = 0.48, p = 0.024 at 10 years) (Fig. 3A,B). Furthermore, seven antibiotics, doxycycline, amoxicillin, azithromycin, ciprofloxacin, levofloxacin, mupirocin, and sulfamethoxazole, and one antiviral drug, valacyclovir, modulated the risk of subsequent comorbidities. Doxycycline consistently increased the risk of ‘cataract NOS’ (adjusted OR = 2.57, p = 0.053 at 2 years/adjusted OR = 1.89, p = 0.058 at 10 years), ‘tear film insuffic NOS’ (adjusted OR = 2.64, p = 0.042 at 5 years/adjusted OR = 2.37, p = 0.050 at 10 years), and ‘nocturia’ (adjusted OR = 3.46, p = 0.010 at 2 years) (Fig. 3B). Amoxicillin, another antibiotic recommended for LD, increased the risk of ‘acute URI NOS’ (adjusted OR = 3.01, p = 6.5E-4 at 2 years/adjusted OR = 2.41, p = 8.4E-4 at 5 years/adjusted OR = 2.60, p = 1.3E-4 at 10 years).

‘Vitamin D deficiency NOS’, common in patients with persistent LD^33^, is a specific form of nutritional deficiency, a comorbidity identified earlier at the broader (CCS-single) level (Fig. 2A,B). Several medications increased the risk of this condition, three at 5 years post-Lyme and five at 10 years. These included two anti-infective drugs, levofloxacin (adjusted OR = 2.68, p = 0.0012 at 10 years) and valacyclovir (adjusted OR = 1.95, p = 0.087 at 5 years/adjusted OR = 2.01, p = 0.065 at 10 years) (Fig. 3A,B).

The risk of developing respiratory disease after LD^34^ was associated with many medications (Fig. 3B). We identified 11 medications that increased risk for these conditions and two that exhibited protective effects. In addition to the three medications reported in SIDER database and amoxicillin and azithromycin above, the medications that conferred increased risk for ‘acute URI NOS’ include an antibiotic, levofloxacin (adjusted OR = 3.18, p = 9.1E-4 at 10 years) and a steroid, methylprednisolone (adjusted OR = 2.14, p = 0.027 at 5 years/adjusted OR = 2.31, p = 0.0077 at 10 years).

### Medications that modulate LD pathophysiology on different timescales

We identified 16 medications associated with disease comorbidities within 5 years post-LD, 81% (13/16) of which overlapped with those identified as associated 10 years post-Lyme by the CCS-single-level categorization. Specifically, five out of six anti-infective drugs, doxycycline, azithromycin, levofloxacin, mupirocin, and valacyclovir, appeared in both timeframes. Moreover, 22 medications were associated with the ICD-9–level disease comorbidities within 5 years after Lyme, 95% (21/22) of which were also identified in the 10-year post-Lyme analysis. Among those 21 medications, five are antibiotics (doxycycline, amoxicillin, azithromycin, ciprofloxacin, mupirocin) and one is an antiviral drug (valacyclovir). The four medications associated with comorbidities exclusively in the 5 years post-Lyme, clavulanate, gabapentin, midazolam, and fentanyl, may impact relatively early Lyme comorbidities (Supplementary Fig. 1).

A total of 17 medications overlapped between the CCS-single and ICD-9 levels in either the 5-year or 10-year time windows. Five of them were anti-infective drugs, namely doxycycline, azithromycin, levofloxacin, mupirocin, and valacyclovir. In the 5-year time window, 16 medications were associated with comorbidities classified by CCS-single-level category, of which 50% (8/16) were also identified at the ICD-9 level. At 10 years post-Lyme diagnosis, we identified 21 significant associations between medications and comorbidities, of which 81% (17/21) were consistent with those identified at the ICD-9 level.

### Survival analysis of first-line medications in propensity-matched populations

By the cross-sectional analysis described above, we demonstrated that certain medications increased risk or protected against disease comorbidities in patients with LD. At higher resolution (i.e., ICD-9 codes) with 10-year follow up, we found that doxycycline, the most commonly used antibiotic for treatment of LD^9^, protected against ‘backache NOS’ and ‘chronic rhinitis’, but increased risk of ‘tear film insuffic NOS’ and ‘cataract NOS’. Another commonly used antibiotic, amoxicillin, was associated with elevated risk of ‘acute URI NOS’.

To clarify the longitudinal effects of doxycycline and amoxicillin, we analyzed these associations by propensity-score-matched survival analyses (Table 3). The doxycycline-treated group was significantly older than the untreated group (P < 0.007), whereas the amoxicillin-treated group was significantly younger than the untreated group (P = 8.7E-4). In addition, doxycycline was prescribed more frequently to male than female patients (P < 0.03). The doxycycline/amoxicillin-treated groups had higher prevalence of certain pre-existing comorbidities and a higher prescription rate of particular medications than the untreated groups (Supplementary Table 4). Moreover, both the doxycycline/amoxicillin treated groups had higher propensity scores than the corresponding untreated groups (P < 0.001).

**Table 3:** Survival analyses of first-line therapeutics for Lyme disease using a propensity-score-matched cohort.

Medication	Disease (ICD9)	ICD9	P value (LogRank)	Hazard Ratio (90% CI)	P value (Cox)
Doxycycline	Tear film insuffic NOS	375.15	6.08E-02	2.65 (1.09–6.45)	7.13E-02
Doxycycline	Cataract NOS	366.9	6.72E-02	1.9 (1.06–3.42)	7.18E-02
Doxycycline	Chronic rhinitis	472.0	3.60E-02	0.49 (0.28–0.87)	3.99E-02
Doxycycline	Backache NOS	724.5	1.67E-02	0.42 (0.23–0.78)	2.03E-02
Amoxicillin	Acute URI NOS	465.9	7.41E-03	2.26 (1.35–3.78)	9.13E-03

**Figure 4:**
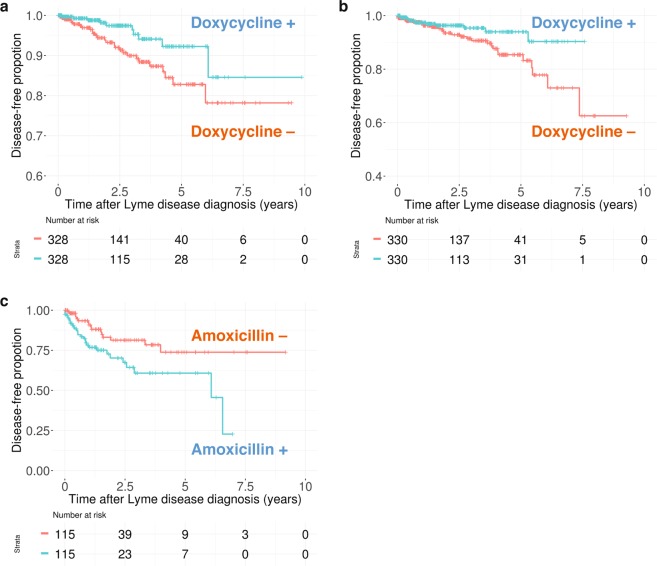
Kaplan–Meier plot of propensity-score-matched survival analysis (**a**) doxycycline–‘backache NOS’ (ICD-9 code: 724.5), (**b**) doxycycline–‘chronic rhinitis’ (472.0), and (**c**) amoxicillin–‘acute URI NOS’ (465.9).

After propensity score matching, the control cohorts were well balanced with the treated groups in terms of observed covariates (Supplementary Table 4). This analysis revealed that the risk of ‘backache NOS’ (Fig. 4a) and ‘chronic rhinitis’ (Fig. 4b) was significantly lower in the doxycycline-treated cohort than in the untreated cohort (HR = 0.42, p = 0.020; HR = 0.49, p = 0.040, respectively; Table 3). Furthermore, Kaplan-Meier curves demonstrated that the cumulative probabilities of remaining free from ‘cataracts NOS’ and ‘tear film insuffic NOS’ were lower among doxycycline-treated patients, but did not reach statistical significance (p = 0.0672 and 0.0608, respectively; Supplementary Fig. 2a,b). Cox regression analysis suggested a trending association between doxycycline usage and increased risk of both ‘cataract NOS’ and ‘tear film insuffic NOS’ (HR = 1.90, p = 0.072; HR = 2.65, p = 0.071). On the other hand, patients prescribed amoxicillin had significantly higher hazard ratios for ‘acute URI NOS’ (HR = 2.26, p = 0.0091; Fig. 4c). Therefore, the effects of doxycycline and amoxicillin revealed by the cross-sectional analysis were confirmed by survival analyses using the propensity score-matched cohort for many of the associations (Table 3).

## Discussion

Proper diagnosis, treatment, and management of Lyme Disease (LD) are difficult for a variety of reasons. In particular, the complex interplay between various treatments and current clinical status, including disease burden, can lead to a wide range of sequelae. This study represents the first data-driven effort to identify clinical factors that affect treatment of LD patients, verified by manual chart review, using large-scale EMR data. In contrast to a one-size-fits-all strategies, our approach may facilitate the personalization of treatment regimens based on the clinical profiles (i.e., disease burden) of affected individuals. This strategic transition is essential in light of the variability in efficacy of antibiotics and the adverse events associated with these treatments (see Supplementary Discussion for further details).

In addition to the co-morbid conditions that present before LD infection (see Supplementary Discussion), we identified conditions that are more likely to be present after a LD diagnosis than beforehand, consistent with the possibility that these diseases are side effects or complications arising from Lyme infection. Many of these associations (e.g., eye-related disorders) are well documented, enhancing our confidence in our results.

The results of this analysis feed into our drug-comorbidity associations network and can be used to inform treatment regimens. For instance, we found that use of steroid medications is associated with increased risk for the symptoms common to Post-Treatment Lyme Disease Syndrome (see Supplementary Discussion). Other findings from our drug-comorbidity network might facilitate personalization of treatment regimens, with more favorable clinical outcomes for patients. For example, several anti-infectious drugs, pain relievers such as diclofenac and hydrocodone, and the anti-allergy medication azelastine were also associated with higher rates of ‘nutritional deficiencies’, suggesting that physicians should consider recommending vitamin supplements for patients receiving these treatments.

Doxycycline is already associated with a range of side effects, including pain, increased pressure inside the skull^35^, and gastrointestinal injury^36^. The nuances of these associations are not well understood. We also found that doxycycline use was associated with lower risk of ‘backache NOS’, often reported a symptom of PTLDS, and lower rates of ‘chronic rhinitis’. This medication showed a trend for risks to cataract (HR = 1.90, p = 0,072), tear film insufficiency (HR = 2.65, p = 0.071) in the survival analyses, which require further validation in both short-term and long-term prospective trials.

This study had several limitations. We did not use a non-LD control cohort for our analyses. A large issue is the relative low LD sample size, which is a consequence of our hospital’s location in the metropolitan area of NYC (https://www.health.ny.gov/statistics/chac/general/g40.htm). Based on the de-identified EMR system we utilized, LD two-tier antibody test results were documented as free text which was masked as PHI content and we do not have capacity to review all charts from EPIC system. As such, due to data availability and sparseness of other clinical variables (e.g., lab test results), we relied on ICD-9 code-based identification of LD patients. However, with a randomly selected patient cohort (N = 50) and physician’s manually chart review, we achieved PPV = 96% with considering three equivocal results, suggesting a strong confidence of identifying true positive LD patients. With our manual chart review approach, however, we were unable to evaluate a false negative rate. As tick bites are most likely to occur in surrounding rural locations in which forests are present, many patients may be initially diagnosed in a different facility, and then come to MSH for follow-up treatment. Another limitation is related to the close proximity of MSH to other medical centers in the area. Specifically, patients may seek treatment at other nearby hospitals, resulting in the loss of valuable information from our EMR system. Finally, because we do not have access to patients’ historic EMR data from outside of MSH, our temporal analyses may not accurately capture the true timeline of acquisition of disease comorbidities. We reported nominal p-values without multiple test correction, because this study is a small-scale, exploratory study. This study will facilitate future work involving cohorts from multiple institutions which will generate more robust findings. We are currently performing an external replication analysis at another academic medical center, and the results of this effort may bolster our conclusions. Furthermore, utilization of a larger LD sample size across multiple medical centers would enable us to analyze combinatorial effects of multiple medications to possible comorbidities, which are also important for management of LD patients. Additionally, we are applying the findings from our current study in order to model explicit, optimal treatment recommendations at the patient level.

Our study is the first to investigate a comprehensive and racially diverse EMR with the aim of discovering the detailed clinical profiles of patients before and after diagnosis of LD. We applied machine learning models and identified a list of medications, including antibiotics that are recommended for treatment, which represent possible risk factors for PTLDS. While we are unable to infer causation from our analyses, further study of the associations we identified could hopefully one day be utilized by physicians to tailor treatments for LD patients based on their current and past physiological state. In addition, we hope to investigate the contributions of genomics and genetic variants to differences pathophysiology. From this work, we hope to enhance not only the success rates of LD treatment, but also to facilitate preemptive strategies for managing high-risk ensuing conditions. All of our findings and recommendations, of course, require further investigation and validation experiments.

## Methods

The study was specifically governed and approved by Institutional Review Board approval at MSH (GCO 15–1805). All patient records were de-identified and analyzed retrospectively, and as such, no informed consent was required. All methods were performed in accordance with the relevant guidelines and regulations.

### Patient population and standardization of clinical terminology

#### Patient cohort

We provide a schematic of our study design, approach, and patient selection criteria in Fig. 1. We utilized Electronic Medical Records (EMRs) from the Mount Sinai Data Warehouse (MSDW), the largest comprehensive EMR system in New York City, which includes data from a racially and ethnically diverse patient base. In this study, we retrieved records from all patients diagnosed with Lyme disease (LD) with the ICD-9 code 088.81 (*n* = 2,134). We restricted the data to records occurring between 2000 and 2015, allowing for up to 15-year follow-up. Finally, we only kept data from patients with defined age, self-reported sex, and self-reported race/ethnicity (referred to as “race” in this manuscript) (*n* = 1,767). For this cohort, there were 930 females (52.6%) and 837 males (47.4%), with an average age of 47.8 ± 19.7. The racial breakdown of the cohort is as follows: 1,201 Caucasian (70.0%), 49 African-American (2.8%), 34 Hispanic/Latino (1.9%), and 483 Others (27.3%). For these patients, we also retrieved all other available clinical variables from EMR, including prescriptions and other disease diagnoses. In total, we compiled 3,936 disease diagnoses and 5,723 prescriptions.

#### Manual chart reviews

Since antibody tests were reported as free text and masked in our de-identified EMR data, a physician performed manual chart reviews for a random subset of the LD cohort (*n* = 50) within the same year of the LD diagnosis to evaluate the accuracy of the phenotyping. The available antibody test was “Lyme Total Antibody with Reflex Western Blot”, coded with CPT code of 86618. Among the 50 reviewed putative LD patients, 45 were reported as positive, two were reported as negative, and three were reported as equivocal results, indicating no clear interpretation of the either positive or negative. The chart review yields a positive predictive value of 0.96 with considering equivocal results. Given the fact that two-tiered antibody diagnostic testing would not detect all true positives and can produce up to 50% false negatives^37^, we were confident in our selection strategy for LD patients in our Mount Sinai EMR.

### Statistical methods and analysis

#### Disease pair temporal directionality

For all patients with LD, we first assessed disease-pair connectivity patterns for comorbid diseases. Specifically, we determined whether the members of each pair exhibited a significant pattern in their temporal order, e.g., whether one preceded the other more often than expected by chance. We performed a cumulative binomial probability test to assess the temporal ordering of the associations between Lyme and all other diseases, assuming a 50% probability of either to occur before the other. We performed the following analysis on both broad and narrow disease categories (see Supplemental Methods).

At the broader level, we analyzed representative CCS-single-level categories because this strategy could enhance signals that might be lost due to small sample size at the ICD-9 level. Second, we performed the analysis using standard ICD-9 codes in order to detect associations at a higher resolution for certain codes that may be more prevalent. Because these comorbid conditions can be either chronic or acute, we performed several iterations of this analysis over different time windows, specifically 2, 5, and 10 years. For each time window, we restricted collection of information for the comorbid diseases in both temporal directions, relative to the date of first Lyme diagnosis. For the 2-year window, for example, we only collected disease data for each patient 2 years before and 2 years after the date of Lyme diagnosis. For the CCS-single- and ICD-9–level analyses, we performed 275 and 3,639 tests for each window, respectively. Last, to determine whether disease pairs with significant temporal directionality were also significantly comorbid, we performed a logistic regression for each pair controlling for age, sex, and self-reported race. The outcome variable in this model was the disease that was shown to occur after the other in the temporal analysis (significant in the binomial assessment).

#### Definition of outcomes and covariates in the statistical model

To discover risk factors or new therapeutic options for LD sequelae, we focused on the new onset of disease comorbidities more than 7 days after the diagnosis of LD. Of the 1,767 LD patients in the overall cohort, we systematically assessed the comorbidities and medication associations for 1,183 patients who were followed up for more than 7 days and had at least one prescription record in MSH’s EMR system. Like our disease-pair temporal directionality analysis, we set time windows of 2, 5, and 10 years. For each patient, we collected the diseases diagnosed within 2, 5, or 10 years after their first Lyme diagnosis date. We also retrieved medications prescribed within 1 year prior to and 2, 5, or 10 years after the first Lyme diagnosis. Outcome comorbidities were defined by ICD-9 code and categorized using CCS-single-level Diagnosis terms.

#### Feature selection

We considered many disease variables, coded by CCS-single-level categories, and medication variables, which were mapped to RxNorm ingredient codes. Accordingly, we adopted a feature selection method, penalized logistic regression with the adaptive LASSO (Eq. 1), to identify variables of the highest relevance that associated with ensuing comorbidities following LD diagnosis. The adaptive LASSO is an extension of the traditional LASSO^38^ that uses coefficient-specific weights^39^. The adaptive LASSO estimator may achieve sparsity and selection consistency for the true model, i.e., correctly identifies the zero and nonzero parameters^40^. Let ${ {\mathcal L} }_{n}(\beta ;Y,X)$ be the negative log-likelihood parametrized by β for a sample of size n. The adaptive LASSO estimator is defined as:

1 $$\hat{\beta }=argmi{n}_{\beta }\{{ {\mathcal L} }_{n}(\beta ;Y,X)+{\lambda }_{n}{{\rm{\Sigma }}}_{j=1}^{d}\widehat{{\omega }_{j}}|{\beta }_{j}|\}$$

where $\widehat{{\omega }_{j}}=|\tilde{{\beta }_{j}}{|}^{-\gamma }$ is a coefficient specific weights vector, and ${\lambda }_{n}$ is a regularization parameter. We set the positive constant γ as 1 according to Zou *et al*.^39^, and obtained $\tilde{\beta }$ by the maximum likelihood estimate of Ridge regression. The ${\lambda }_{n}\,$value for minimum AUC was chosen by 10-fold cross validation. We used the R package *glmnet*^41^ for these penalized regressions.

#### Logistic regression model

We used odds ratio (OR) from logistic regression (Eq. 2) to assess the risk of future comorbidity progression on each medication taken (i.e. either increased risk or protective effect). We analyzed the pairs of outcome disease comorbidity and the medications that were selected by the adaptive LASSO. In this model, we adjusted for age, sex, self-reported race, and the follow-up time frame.

2 $$log(\frac{P}{1-P})={\beta }_{0}+{\beta }_{m}medication+{\beta }_{a}age+{\beta }_{g}gender+{\beta }_{r}race+{\beta }_{p}observered\,period$$

where *P* is the probability of a disease, medication is a binary variable, age is a continuous parameter, gender is a binary variable (Female/Male); race is a categorical variable (Caucasian, African American, Hispanic/Latino, or Other), and observed period is a continuous parameter. $\beta$ coefficients for each covariate represent the effect size when controlling for all others.

#### Propensity score matching

To control for potential confounding factors due to imbalances of clinical characteristics, not limited to age and gender, we analyzed the temporal effects of medications after the propensity score matching to select an appropriate control cohort for the targeted case cohort^42^. Thus, we created comparable cohorts, consisting of groups treated or untreated with a targeted medication, based on a set of covariates at the baseline time point, i.e., time zero for each patient. The baseline time point was defined as the first prescription day of the targeted medication or 7 days after LD diagnosis, whichever was later, because we observed disease comorbidities for more than 7 days after LD diagnosis.

The propensity scores of targeted prescriptions were predicted by a logistic regression model, including other significant medications and disease confounders selected by the adaptive LASSO with a 10-year time window, with patient demographics as covariates. Each patient prescribed a given medication was matched to a corresponding comparison patient (1:1 ratio) by nearest-neighbor matching. For instance, we analyzed association between doxycycline and ‘backache Not Otherwise Specified (NOS)’ (ICD-9 code: 724.5), ‘chronic rhinitis’ (472.0), ‘tear film insufficiency (insuffic) NOS (375.15)’, and ‘cataract NOS (366.9)’, and between amoxicillin and ‘acute upper respiratory infection (URI) NOS (465.9)’. A total of 328, 330, 358, 370, and 115 subjects were selected for each medication-comorbidity pair in the propensity score-matched treated/untreated group. The R package *MatchIt*^43^ was used for propensity score matching.

#### Survival analysis

We generated survival curves by the Kaplan–Meier method and examined differences in survival among subgroups by the log-rank test, with propensity score matching of cases and controls. We calculated hazard ratios using Cox proportional hazards models:

3 $$h(t)={h}_{0}(t)\exp ({\beta }_{m}medication)$$

where *h*(*t*) is the expected hazard at time t, *h*_0_(*t*) is the baseline hazard, and medication is a binary variable. We verified the proportional hazards assumption by confirming that Schoenfeld residuals are independent of time (Schoenfeld test p > 0.1). We used the R packages *survival* and *survminer* for the survival analysis.

## Supplementary information

Supplementary Materials

Supplementary Table 1

Supplementary Table 2

Supplementary Table 3

Supplementary Table 4
