# Supplementary material for: Lyme Disease Patient Trajectories Learned from Electronic Medical Data for Stratification of Disease Risk and Therapeutic Response
Source: Sci Rep. 2019 Mar 14;9:4460. doi: 10.1038/s41598-019-41128-x (PMC6418311; doi:10.1038/s41598-019-41128-x)
Supplement: Supplementary file 1 — Supplementary Materials [file 41598_2019_41128_MOESM1_ESM.docx]

**Lyme Disease Patient Trajectories Learned from Electronic Medical Data for Stratification of Disease Risk and Therapeutic Response**

Osamu Ichikawa^1,2#^, Benjamin S. Glicksberg^1,3#^, Nicholas Genes^4^, Brian A. Kidd^1^, Li Li^1,5*^, Joel T. Dudley^1*^

1. Department of Genetics and Genomic Sciences, Institute for Next Generation Healthcare, Icahn School of Medicine at Mount Sinai, 770 Lexington Ave., New York, NY 10065.
2. Drug Research Division, Sumitomo Dainippon Pharma. Co. Ltd., 3-1-98 Kasugade-naka, Konohana-ku, Osaka, 554-0022, Japan.
3. Bakar Computational Health Science Institute, University of California, 550 16^th^ St, San Francisco, California, 94158.
4. Department of Emergency Medicine, Icahn School of Medicine at Mount Sinai, One Gustave L. Levy Place Box 1620 New York, NY 1002
5. Sema4, a Mount Sinai Venture, Stamford, Connecticut, 06902

# These authors contributed equally to this work

* Correspondence and requests for materials should be address to

Dr. Li Li ([li.li@mssm.edu](mailto:li.li@mssm.edu)) or Dr. Joel Dudley ([joel.dudley@mssm.edu](mailto:joel.dudley@mssm.edu))

Institute for Next Generation Healthcare

Icahn School of Medicine at Mount Sinai

770 Lexington Ave.

15^th^ Floor

New York, NY 10065, USA

**Keywords:** Electronic Medical Records; Lyme disease; Machine Learning; Post-Treatment Lyme Disease Syndrome**;** Precision Medicine.

**SUPPLEMENTAL MATERIALS**

**Supplementary Background**

***Issues surrounding Lyme Disease laboratory testing***

Accurate and precise diagnosis of LD presents several challenges. Typically, laboratory testing of LD follows identification of cutaneous manifestations from visual inspection but these manifestations are not always present. Current guidelines recommend serologic testing, a two-phase process consisting of an enzyme-linked immunosorbent assay and IgM western blot within 30 days of symptom onset or IgG confirmation by Western blot after 30 days from symptom occurs^6-8^. Even together, this diagnostic strategy has poor sensitivity, particularly during the acute phase, with false-negative rates of up to 50%^9^ . Other laboratory methods are specific for particular manifestations, e.g., testing of CSF for central nervous system involvement. Recent work has shown that incorporation of data from various wearable devices can detect early signs of LD and associated inflammatory responses^10^. For example, variations in peripheral capillary oxygen saturation (SpO2), a marker associated with physiological macro-phenotypes such as fatigue, can be measured by portable biosensors to facilitate more accurate and rapid LD diagnosis from variations in these measurements and could be economically feasible for widespread use in the future. Currently, however, clinicians still have to rely on traditional measures to diagnose patients. Furthermore, comorbid conditions can interfere with both diagnosis and treatment. For instance, other infections can be concurrently transmitted with LD ^11^, making differential diagnosis even more difficult and sometimes requiring specialized, alternative treatment strategies.

***Risk factors for Lyme Disease***

An investigation of risk factors for LD infection, such as behavioral and environmental risk factors, revealed that LD–positive serology is significantly associated with clinical and demographic features such as previous self-reported LD diagnosis and age, behavioral factors such as wearing protective clothing, and geographic/environmental factors such as shrub edge density in property location ^12^. Another study evaluated the risks to individuals based on geographical features such as the suitability of the local habitat for ticks ^13^.

***Lyme Disease incidence rates in New York City***

In this study, we leverage an EMR data set representing over five million unique patients of diverse racial and ethnic backgrounds collected from a large academic medical center in New York City. Although not itself located adjacent to a wooded area, Mount Sinai Hospital (MSH) caters to patients from all over the state, including residents who travel to endemic areas. New York is one of the 14 states reporting the vast majority of LD cases, and in 2017 had an incidence rate of 16.4 per 100,000 individuals (CDC), one of the highest in the country. The New York State Department of Health reports the incidence rate for LD in New York State to be somewhat higher at 38.1 per 100,000 individuals as of 2015 (<https://www.health.ny.gov/statistics/chac/general/g40.htm>).

**Supplemental Methods**

- ***Clinical sources and term standardization***
- We categorized diseases using the Clinical Classifications Software (CCS) for ICD-9 diagnosis codes, developed by AHRQ ^14^, which aggregates and characterizes more than 14,000 ICD-9 codes into broader coherent disease categories. This strategy helps to avoid sample size limitation as a result of using ICD-9 codes alone. For categorization, we used the ‘Single-Level Diagnosis’ (CCS-single) level, which has a total of 283 different categories. We standardized medication data by mapping to the RxNorm ontology^15^. Specifically, we mapped these terms to ingredient codes, yielding 793 normalized medications. In consideration for statistical power, we required sample size of > 20 patients for calculations of the significance of disease directionality and disease–medication association.

**Supplementary Discussion**

In this study, we performed analyses on clinical data for 2,134 LD patients identified in our EMR. Based on our overall EMR size of over 2 million unique patients with at least one clinic visit and the regional LD rate of 9.9 per 100,000 (https://www.health.ny.gov/statistics/chac/general/g40.htm; 2013-2015 average) reported in the five boroughs, we would expect 2,970 LD cases (for the 15 years of patient data we have in our EMR). Given the fact that LD prevalence has been increasing in the recent years, we believe 2,134 LD patients is reasonable. We found all significant comorbidities of patients with LD before and after their Lyme infections, which included many associations that warrant further exploration. We identified an association between HIV infection and LD. Specifically, our data showed that patients with HIV almost exclusively developed LD subsequent to the HIV diagnosis, suggesting that immune system alteration increases the risk of LD. A handful of case reports have indicated that HIV-positive immunocompromised patients develop more severe Lyme complications following infection ^16-18^. Although the specific immunological mechanisms driving this connection remain unclear, it seems reasonable to speculate that immunosuppression plays an important role ^19^. Additionally, our analysis identified co-morbid conditions that often present before LD infection. Although LD requires contact with the bacteria, certain physiological properties make individuals more or less susceptible to infection. For example, we found that individuals categorized as having ‘disorders of lipid metabolism’ were more likely have LD infection in the future. *Borrelia burgdorferi* requires cholesterol for growth; researchers have found that apolipoprotein E (apoE)-deficient and low-density lipoprotein receptor (LDLR)-deficient mice, which have high levels amounts of serum cholesterol, are more susceptible than wild-type mice to pathogenesis induced by this bacterium ^20^. Additionally, patients with hypercholesterolemia could increase susceptibility to trigger tick bite for this vector borne disease due to body heat, CO_2_, and moisture which are key attractants similarly to mosquitoes ^21^ ^22^, but this is beyond the scope of this analysis and needs to be further investigated. We believe that the results from drug-comorbidity associations network can be used to help direct treatment regiments. These agents prevent the development of many complications associated with LD, however, long-term exposure to these medications pose significant complications. Indeed, it is possible that even acute use (usually a month) of these treatments is associated with long-term complications of LD that yet to be determined. Even when treated, up to 20% of patients develop Post-Treatment Lyme Disease Syndrome (PTLDS), in which symptoms including fatigue or muscle pain last for months or years. Although the etiology of PTLDS is not yet known, better tailoring of treatment strategies to an individual’s phenotypic profile could prevent or modulate the risk of developing these symptoms. We identified the comorbidities matching the symptoms aligned with PTLDS, including chronic pain (Figures 3a and 3b). Notably in this regard, we also found that usage of steroid medications increases the risk of many symptoms common to PTLDS. Consistent with this, corticosteroid use is associated with poor outcomes for LD patients ^23^. In particular, we found that prednisone was associated with elevated risk for ‘backache NOS’, ‘pain in limb’ and ‘other abnormal glucose’, defined at the ICD-9 level. Use of fluticasone, mometasone, and methylprednisolone were associated with elevated risk for upper respiratory diseases, spondylosis or backache. Steroids, which suppress patients’ immune systems, might be particularly harmful to LD patients, allowing the bacteria to grow, rather than attacking the infections. These findings suggest that steroid use should be limited in LD patients, and that patients exposed to these drugs should be monitored carefully for complications.

Additionally, the risk of respiratory-related conditions after LD was associated with many medications. In one case study, secondary adult respiratory distress syndrome caused the death of a patient affected with LD during the course of her 2-month treatment ^24^. The patient did not respond to conventional treatments, including antibiotics, salicylates, and steroids. In our study, we identified medications that are associated with elevated risk of respiratory-related diseases. Specifically, we found that the antibiotics amoxicillin, levofloxacin, and azithromycin all conferred increased risk of ‘acute URI NOS’. In addition to the known risks of two steroids, prednisolone, and mometasone, reported in the SIDER database (S table 3), we found that another steroid, methylprednisolone, was also associated with increased risk for this disease.

**Supplementary Table Legends**

**Supplementary Table S1** All diseases associated with Lyme, by ICD-9 category (binomial test p value < 0.1). Mapping ICD-9 short descriptions to ICD-9 full descriptions were also provided in this table.

**Supplementary Table S2** Medications predicted to modulate risk of disease comorbidities, by CCS-single-level category (p value < 0.1).

**Supplementary Table S3** Medications predicted to modulate risk of disease comorbidities, by ICD-9 code (p value < 0.1).

**Supplementary Table S4** The balance of covariates before and after propensity score matching.

**Supplementary Figures**

**Supplementary Figure 1** Venn diagram of the medications that significantly associated with at least one disease comorbidity in the 5- and 10-year time windows. (a) CCS-single-level categories. (b) ICD-9 level.

**Supplementary Figure 2** Kaplan–Meier plot of propensity-score-matched survival analysis (a) doxycycline–‘cataract NOS’ (366.9) and (b) doxycycline–‘tear film insuffic NOS’ (375.15).

**Supplementary References**

1 Wormser, G. P. *et al.* Borrelia burgdorferi genotype predicts the capacity for hematogenous dissemination during early Lyme disease. *J Infect Dis* **198**, 1358-1364, doi:10.1086/592279 (2008).

2 Hanincova, K. *et al.* Multilocus sequence typing of Borrelia burgdorferi suggests existence of lineages with differential pathogenic properties in humans. *PLoS One* **8**, e73066, doi:10.1371/journal.pone.0073066 (2013).

3 Koedel, U., Fingerle, V. & Pfister, H. W. Lyme neuroborreliosis-epidemiology, diagnosis and management. *Nat Rev Neurol* **11**, 446-456, doi:10.1038/nrneurol.2015.121 (2015).

4 Klempner, M. S. *et al.* Two controlled trials of antibiotic treatment in patients with persistent symptoms and a history of Lyme disease. *N Engl J Med* **345**, 85-92, doi:10.1056/NEJM200107123450202 (2001).

5 Fallon, B. A. *et al.* A randomized, placebo-controlled trial of repeated IV antibiotic therapy for Lyme encephalopathy. *Neurology* **70**, 992-1003, doi:10.1212/01.WNL.0000284604.61160.2d (2008).

6 Engstrom, S. M., Shoop, E. & Johnson, R. C. Immunoblot interpretation criteria for serodiagnosis of early Lyme disease. *J Clin Microbiol* **33**, 419-427 (1995).

7 Dressler, F., Whalen, J. A., Reinhardt, B. N. & Steere, A. C. Western blotting in the serodiagnosis of Lyme disease. *J Infect Dis* **167**, 392-400 (1993).

8 Fallon, B. A., Pavlicova, M., Coffino, S. W. & Brenner, C. A comparison of lyme disease serologic test results from 4 laboratories in patients with persistent symptoms after antibiotic treatment. *Clin Infect Dis* **59**, 1705-1710, doi:10.1093/cid/ciu703 (2014).

9 Marques, A. R. Laboratory diagnosis of Lyme disease: advances and challenges. *Infect Dis Clin North Am* **29**, 295-307, doi:10.1016/j.idc.2015.02.005 (2015).

10 Li, X. *et al.* Digital Health: Tracking Physiomes and Activity Using Wearable Biosensors Reveals Useful Health-Related Information. *PLoS Biol* **15**, e2001402, doi:10.1371/journal.pbio.2001402 (2017).

11 Berghoff, W. Chronic Lyme Disease and Co-infections: Differential Diagnosis. *Open Neurol J* **6**, 158-178, doi:10.2174/1874205X01206010158 (2012).

12 Finch, C. *et al.* Integrated assessment of behavioral and environmental risk factors for Lyme disease infection on Block Island, Rhode Island. *PLoS One* **9**, e84758, doi:10.1371/journal.pone.0084758 (2014).

13 Guerra, M. *et al.* Predicting the risk of Lyme disease: habitat suitability for Ixodes scapularis in the north central United States. *Emerg Infect Dis* **8**, 289-297, doi:10.3201/eid0803.010166 (2002).

14 Healthcare Cost and Utilization Project (HCUP). (Agency for Healthcare Research and Quality, 2017).

15 Nelson, S. J., Zeng, K., Kilbourne, J., Powell, T. & Moore, R. Normalized names for clinical drugs: RxNorm at 6 years. *J Am Med Inform Assoc* **18**, 441-448, doi:10.1136/amiajnl-2011-000116 (2011).

16 Gugliotta, J. L., Goethert, H. K., Berardi, V. P. & Telford, S. R., 3rd. Meningoencephalitis from Borrelia miyamotoi in an immunocompromised patient. *N Engl J Med* **368**, 240-245, doi:10.1056/NEJMoa1209039 (2013).

17 Bremell, D., Sall, C., Gisslen, M. & Hagberg, L. Lyme neuroborreliosis in HIV-1 positive men successfully treated with oral doxycycline: a case series and literature review. *J Med Case Rep* **5**, 465, doi:10.1186/1752-1947-5-465 (2011).

18 van Burgel, N. D., Oosterloo, M., Kroon, F. P. & van Dam, A. P. Severe course of Lyme neuroborreliosis in an HIV-1 positive patient; case report and review of the literature. *BMC Neurol* **10**, 117, doi:10.1186/1471-2377-10-117 (2010).

19 Elsner, R. A., Hastey, C. J., Olsen, K. J. & Baumgarth, N. Suppression of Long-Lived Humoral Immunity Following Borrelia burgdorferi Infection. *PLoS Pathog* **11**, e1004976, doi:10.1371/journal.ppat.1004976 (2015).

20 Toledo, A., Monzon, J. D., Coleman, J. L., Garcia-Monco, J. C. & Benach, J. L. Hypercholesterolemia and ApoE deficiency result in severe infection with Lyme disease and relapsing-fever Borrelia. *Proc Natl Acad Sci U S A* **112**, 5491-5496, doi:10.1073/pnas.1502561112 (2015).

21 Enserink, M. What mosquitoes want: secrets of host attraction. *Science* **298**, 90-92, doi:10.1126/science.298.5591.90 (2002).

22 Kim, Y. M., Kim, S., Cheong, H. K., Ahn, B. & Choi, K. Effects of heat wave on body temperature and blood pressure in the poor and elderly. *Environ Health Toxicol* **27**, e2012013, doi:10.5620/eht.2012.27.e2012013 (2012).

23 Jowett, N., Gaudin, R. A., Banks, C. A. & Hadlock, T. A. Steroid use in Lyme disease-associated facial palsy is associated with worse long-term outcomes. *Laryngoscope* **127**, 1451-1458, doi:10.1002/lary.26273 (2017).

24 Kirsch, M. *et al.* Fatal adult respiratory distress syndrome in a patient with Lyme disease. *JAMA* **259**, 2737-2739 (1988).
